# Extended versus inpatient thromboprophylaxis with heparins following major open abdominopelvic surgery for malignancy: a systematic review of efficacy and safety

**DOI:** 10.1186/s13741-020-0137-8

**Published:** 2020-03-03

**Authors:** B. Heijkoop, S. Nadi, D. Spernat, G. Kiroff

**Affiliations:** 1 The Queen Elizabeth Hospital, Woodville, SA Australia; 2 Discipline of Surgery, The University of Adelaide, Adelaide, Australia; 3 Research and Evaluation, Incorporating ASERNIP-S, Royal Australasian College of Surgeons, Adelaide, Australia

**Keywords:** Venous thromboembolism, Malignancy, Surgery, Heparin, Prophylaxis

## Abstract

**Background:**

Patients undergoing open abdominopelvic procedures for malignancy are at high risk of postoperative venous thromboembolism (VTE). This risk can be mitigated with prophylaxis; however, optimum duration in this population remains unknown. Our objective was to conduct a systematic review of contemporary literature on the use of heparin thromboprophylaxis following major open pelvic surgery for malignancy, comparing the efficacy and safety of extended duration to inpatient treatment.

**Methods:**

A study protocol describing search strategy and inclusion and exclusion criteria was developed and registered with PROSPERO. A literature review was conducted in accordance with the protocol.

**Results:**

Literature review identified only 4 studies directly comparing extended and inpatient duration prophylaxis, with a combined population of 3198 and 3135 patients for VTE rate and bleeding events, respectively. Despite many studies reporting lower VTE rates in patients receiving extended prophylaxis, no statistically significant difference in rates of postoperative VTE (*p* = 0.18) or bleeding complications (*p* = 0.43) was identified between patients receiving extended duration prophylaxis and those receiving inpatient only prophylaxis.

**Conclusion:**

On the review of contemporary literature, no significant difference was found in rates of postoperative VTE or bleeding complications between patients receiving extended duration heparin VTE prophylaxis and those receiving inpatient prophylaxis after open abdominopelvic surgery for malignancy.

This raises the question of how extended duration prophylaxis has become common practice in this population, and whether this needs to be re-evaluated.

## Background

Venous thromboembolism (VTE) is a common postoperative complication associated with significant morbidity and mortality (American Urological Association 2013). A major risk factor for VTE is type of surgery, with patients undergoing major oncological surgery or pelvic surgery being at significant risk (Violetti et al. 2016). These patients frequently also have additional non-modifiable risk factors for VTE including advanced age, limited mobility, previous VTE, or hereditary pro-thrombotic disorders. However, these risks can be mitigated by using prophylaxis. Best practice guidelines, including the current *British Journal of Urology* (BJUI) (Violetti et al. 2016) recommendation and those previously produced by American Urological Association (AUA) (American Urological Association 2013), recommend the use of low molecular weight heparin (enoxaparin) or unfractionated heparin in patients who are at high risk of VTE although they do not specify an exact quantified recommended duration of treatment.

However, despite consensus that the risk of VTE extends for a significant period postoperatively, to date, literature reviews have found insufficient evidence to determine an exact time frame for this, and consequently have not been able to make an evidence-based recommendation for the optimum duration of prophylaxis (Violetti et al. 2016). In addition, there does not appear to be a consistent pattern of the use of postoperative pharmacological VTE prophylaxis in pelvic oncological surgery patients.

As with all interventions, the benefit must be weighed against the potential for adverse events. Known complications of pharmacological DVT/VTE prophylaxis include both major and minor hemorrhage, thrombocytopenia, elevation of serum aminotransferases, infection associated with hematoma, hypersensitivity reactions, and local reactions (Australian Medicines Handbook 2019). With increased duration of prophylaxis, there will be an increase in prophylaxis-related adverse events, up to a point where these outweigh any ongoing benefit of the prophylaxis—again, at what duration of prophylaxis this point is reached remains unclear. Furthermore, extending the duration of VTE prophylaxis beyond what is required adds an economic burden to the health care system.

Consequently, further investigation is warranted to define the optimum duration of postoperative pharmacological VTE prophylaxis with heparin following major pelvic oncological surgery to reduce the risk of VTE without disproportionately increasing the risk of heparin-associated complications. Identifying this and making an evidence-based recommendation would enable all pelvic oncological surgery patients to receive standardized best practice postoperative pharmacological VTE prophylaxis.

In this article, we conduct a meta-analysis of inpatient versus extended duration VTE prophylaxis in patients undergoing pelvic surgery for malignancy. Extended use was defined as any continuation of pharmacological VTE prophylaxis after discharge from the index hospital admission, with inpatient use defined as the use of pharmacological VTE prophylaxis for the majority of the index hospital inpatient admission.

## Methods

A study protocol describing search strategy and inclusion and exclusion criteria was developed and registered with PROSPERO (CRD42018068961).

In accordance with the protocol, literature search was conducted in PubMed, EMBASE, and Cochrane databases. Search terms included medical subject headings (MeSH) and keywords combined by Boolean operators: aspirin, dalteparin, enoxaparin, warfarin, heparin, low molecular weight heparin, abdominal neoplasm, pelvic neoplasm, prostate cancer, bladder cancer, ureteric cancer, urethral cancer, ovarian cancer, cervical cancer, and uterine cancer.

Search results were screened by the primary author and initially shortlisted for inclusion or discarded based on the relevance of the title to the protocol. Of those shortlisted by title, the abstract was reviewed and the papers were returned to the shortlist or discarded based on the relevance of the abstract. Those included or unclear based on the abstract proceeded to review of the entire article by both the primary author and second peer reviewer, who independently documented if they would include or discard the article. Disagreement was resolved by discussion between the reviewers.

Studies identified were eligible for inclusion in the review if they met inclusion criteria of being English language studies of adult patients published within the last 10 years at the time of literature search. All National Health and Medical Research Council (NHMRC) levels of evidence (I–V) were included as it was felt the inclusion of case series and case reports remained important as a means of capturing reporting of adverse events. Non-English language papers, pediatric populations, non-operative or exclusively laparoscopic surgical populations, and animal studies were excluded. While other antithrombotic agents (warfarin, aspirin) were included in the initial search terms with the aim of capturing a broad range of literature on postoperative thromboprophylaxis, a decision was made to assess only papers regarding heparin thromboprophylaxis and those utilizing other forms of thromboprophylaxis were excluded.

The intervention of interest was the use of extended pharmacological VTE prophylaxis with any form of heparin postoperatively following major open abdominal or pelvic surgery for malignancy, with the comparator being inpatient use of heparin pharmacological VTE prophylaxis in this population. Extended use was defined as any continuation of pharmacological VTE prophylaxis after discharge from the index hospital admission, with inpatient use defined as the use of pharmacological VTE prophylaxis for the majority of the index hospital inpatient admission. The primary outcome was the number of clinically evident VTE events objectively confirmed on investigation with ultrasound (US), computed tomography pulmonary angiography (CTPA), or nuclear ventilation/perfusion (V/Q) scan in patients treated with prophylactic heparin following major pelvic surgery for malignancy. Secondary outcomes included adverse events attributable to the use of pharmacological VTE prophylaxis such as bleeding, hematoma, thrombocytopenia, drug reaction, and in association with identified VTE events: length of stay, ICU admission, or readmission to hospital following discharge.

Assumptions made included that all patients received an appropriate dose of pharmacological prophylaxis for their body habitus and pre-existing conditions such as renal impairment, that patients receiving pharmacological prophylaxis also received appropriate non-pharmacological VTE prophylaxis (such as compression stockings, sequential compression devices), and that potential VTE events not diagnosed on US, CTPA, or V/Q scan are of sufficiently insignificant impact on the individual’s recovery as to be irrelevant to the outcomes of the study. Finally, despite the inherently variable length of individual patients’ inpatient admission, inpatient use of prophylaxis was considered as a single duration as the inpatient hospital setting was considered to have a significant impact on patient’s VTE risk due to altered mobility from baseline. Likewise, while individual extended prophylaxis regimes varied in their exact duration, these were considered as a single group.

Following final identification of included articles and data extraction, “Revman” (Review Manager (REVMAN) [computer program] 2014) software was used to directly compare appropriate articles and produce forest plots of these comparisons. Narrative review of remaining articles not appropriate for direct comparison was then performed.

## Results

Final database search using all fields OR MeSH terms for; aspirin, dalteparin, enoxaparin, warfarin, heparin, low molecular weight heparin AND All fields OR MeSH terms for; abdominal neoplasm, pelvic neoplasm, prostate cancer, bladder cancer, ureteric cancer, urethral cancer, ovarian cancer, cervical cancer, uterine cancer identified 3381 articles.

**Fig. 1:**
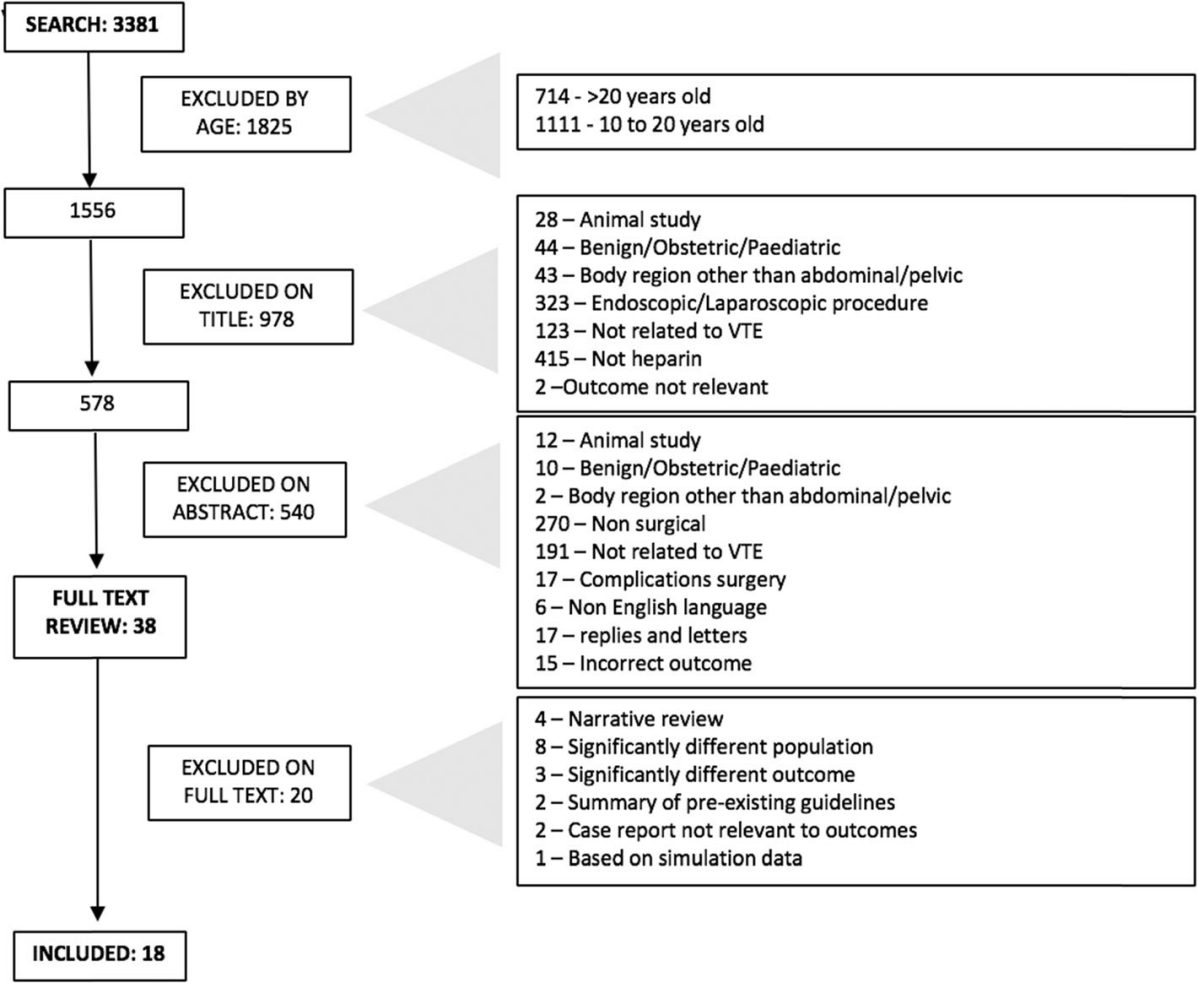
Flow diagram summarizing exclusions on literature search

Of these 3381 articles, 1825 were excluded by age of publication, with a further 977 excluded on review of title and 540 excluded on review of abstract, leaving a total of 38 articles for full text review. The exclusion process is summarized in Fig. 1.

Following full text review by both reviewers, a total of 18 articles met inclusion criteria. Of the 20 excluded on full text review, reasons for exclusion included narrative reviews (4), significant differences in population (8), or outcome (3), as well as 2 articles which simply summarized pre-existing guidelines, 2 case reports not relevant to complications of anticoagulation, and 1 article that was based on a simulation only.

Two pre-existing systematic reviews were identified—Fagaranasu et al. (2016) and Akl et al. (2008). The characteristics of these can be summarized in Table 1.

**Table 1:** Summary of characteristics of two pre-existing reviews (Fagaranasu et al. 2016; Akl et al. 2008)

	Fagaranasu et al. (SR1)	Akl et al. (SR2)
Title	Role of extended thromboprophylaxis after abdominal and pelvic surgery in cancer patients: a systematic review and meta-analysis	Extended perioperative thromboprophylaxis in patients with cancer, a systematic review
Journal	Ann Surg Oncol	Cellular proteolysis and oncology
Year	2016	2008
Country	Canada	USA
Funding	Did not comment	“Institutional support” + one author funded by a European Commission
Time period included	Inception of database—May 2015	Did not comment
Databases searched	- Cochrane Central Register of Controlled Trials - MEDLINE - EMBASE	- Cochrane Register of Controlled Trials (Jan. 2007) - MEDLINE - EMBASE - ISI the Web of Science - CENTRAL
MeSH terms used	- Abdominal surgery - Pelvic surgery - Thromboprophylaxis	Not listed
Supplementary materials	- Abstracts from hematology oncology conferences - Clinical trial registries - Manual search of reference lists - Screening health technology assessments	- Hand searching of conference proceedings - Review of reference lists - Related article feature in PubMed
Population	- Adult (> 18 years) patients receiving thromboprophylaxis with low molecular weight heparin after abdominal or pelvic cancer surgery	Adult patients with abdominal cancer undergoing abdominal surgery
Inclusion criteria	- Randomized clinical trial or prospective observational cohort comparing extended thromboprophylaxis (2–6/52 postop) with conventional thromboprophylaxis (< 2/52 postop) - Use of thromboprophylaxis with low molecular weight heparin - All VTE outcomes objectively diagnosed using US/CTPA/VQ scan - Included asymptomatic objectively diagnosed VTE - Study reported at least one of DVT, PE, mortality, and major bleeding - Adult (> 18) patients	- Randomized controlled trials assessing all-cause mortality, symptomatic DVT, pulmonary embolism, major bleeding, minor bleeding, injection site hematoma, and heparin induced thrombocytopenia - Follow-up rate equal to or greater than 80% for the outcome under consideration
Quality assessment tool	Newcastle-Ottawa scale	GRADE
Number of papers identified; included	2763 papers identified 32 full text review 7 eligible included studies (3 randomized controlled trials, 4 observational)	3986 papers identified 3 eligible included studies
Outcomes-efficacy	1) All VTE: extended thromboprophylaxis significantly reduced incidence of all VTE compared with conventional thromboprophylaxis (2.6% versus 5.6% risk ratio (RR) 0.44, confidence interval (CI) 0.28–0.70, number needed to treat (NNT) 39) 2) Proximal DVT: incidence significantly lower in extended thromboprophylaxis group (1.4% versus 2.8% CI 0.23–0.91, NNT 71) 3) Distal DVT: results did not reach statistical significance 4) PE: no statistically significant difference between PE incidence in the two groups	1) Symptomatic DVT: none had analyzable data 2) Asymptomatic DVT: only one study with sufficient follow-up rate—statistically significant difference at 4 weeks postop 3) PE: only reported in one study, insufficient follow-up rate for criteria
Outcomes-safety	1) Major bleeding: no statistically significant difference observed 2) All-cause mortality: overall mortality similar 4.2% versus 3.6% RR 0.79, CI 0.47–1.33, NNT 167	1) All-cause mortality: only reported in one study, with no statistically significant difference at 3 or 12 months. 2) Bleeding: only reported in one study—no statistically significant difference at 4 weeks or 3 months postoperatively for either major or minor bleeding
Conclusions	Extended thromboprophylaxis significantly reduces the overall incidence of VTE and proximal DVT without increasing the risk of major bleeding.	There is limited and low-quality evidence that extended duration of low molecular weight heparin for perioperative thromboprophylaxis reduces DVT in patients with cancer undergoing major abdominal or pelvic surgery. More and better quality evidence is needed to justify extended regimens.
Acknowledged limitations of review	- Only 3 randomized controlled trials identified - Heterogeneity from inclusion of different types of surgeries, 3 studies allowed inclusion laparoscopic interventions - Insufficient data on specific cancer types and stages; consequently, individualized recommendations cannot be derived from data - Open label nature of some included studies; associated bias in patient symptom reporting and physician suspicion of VTE	- Restriction of electronic search strategy to patients with cancer (potential missed studies with subgroups of cancer patients) - Limited number and low quality of included studies (included small sample size, high loss to follow up, focus on asymptomatic DVT)
Strengths of review	- Comprehensive search - Inclusion all major prospective studies to date assessing ETP after major abdominal and pelvic cancer surgery - All VTE objectively diagnosed - Outcomes between studies reasonably similar - Low statistical heterogeneity	- Use of Cochrane collaboration methodology - Priori definition of outcomes - GRADE approach to evaluate quality of evidence

Of the 18 included articles, only 4 directly compared inpatient and extended duration VTE prophylaxis making them suitable for statistical analysis. These were Schomburg et al. (2018), Samama et al. (2014), Holwell et al. (2014), and Kakkar et al. (2010). Furthermore, the only outcome consistently reported across all 4 of these studies was total VTE rate. Kakkar et al. and Samama et al. both reported DVT rate as a subgroup of VTE. Three (Kakkar et al., Samama et al., and Schomburg et al.) reported bleeding complications; however, only Kakkar et al. and Samama et al. further reported subgroups of critical and fatal bleeding.

**Fig. 2:**
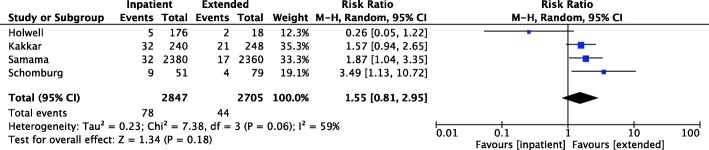
Forest plot illustrating direct comparison of total VTE rate

Regarding the primary outcome of VTE events, there was a non-significant risk reduction (risk ratio 1.55, CI 0.81–2.95) in total VTE rate. Moderate heterogeneity between the studies included in the assessment of total VTE rate was observed (*I*^2^ = 59%). This is illustrated in Fig. 2. No subgroups (DVT rate, PE rate) were assessed by more than two included articles.

**Fig. 3:**
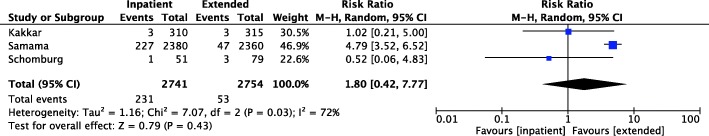
Forest plot illustrating direct comparison of bleeding events (any)

The only secondary outcome assessed across more than one of the direct comparison articles was the total rate of any bleeding complication, in Kakkar et al. (2010), Samama et al. (2014), and Schomburg et al. (2018). This result was also non-significant (*p* = 0.43), and there was a larger degree of heterogeneity (*I*^2^ = 72%) seen between the results with only Samama et al. (2014) independently reaching statistical significance. This is illustrated in Fig. 3.

While additional outcomes including DVT rate, bleeding in a critical organ, and fatal bleeding were covered by both the results in Kakkar et al. and Samama et al., we did not feel it was appropriate to directly compare two papers only.

Of the remaining 11 included articles, 8 reported outcomes relevant to the efficacy of heparin VTE prophylaxis while 1 reported outcomes relevant to the safety of heparin use as VTE prophylaxis in patients following open abdominal or pelvic surgery for malignancy. Two reported both efficacy and safety outcomes. None directly compared inpatient and extended duration prophylaxis.

Once again, the only efficacy outcome that was consistently reported across included studies was total VTE rate. Some studies did analyze subgroups of DVT versus PE rates and symptomatic versus asymptomatic VTE. Chandra et al. (2013), Sanderson et al. (2011), Mohn et al. (2011), and Varpe et al. (2009) all published cohort studies on the efficacy of inpatient heparin VTE prophylaxis following colorectal procedures, with all reporting low VTE rates in these patients with a range between 0.6 (Varpe et al. 2009) and 1.35% (Mohn et al. 2011). While these papers were comparable not only in their population (colorectal cancer patients) and length of follow-up (1 to 3 months), all were limited by a small sample size.

Klimowicz White et al. (2015), Sun et al. (2015), Jeong et al. (2010), and Beyer et al. (2009) also all included a cohort of patients receiving inpatient heparin, with comparisons made to mechanical prophylaxis and no prophylaxis, as well as comparison to therapeutic enoxaparin in Schmitges et al. (2012) and warfarin anticoagulation by Sun et al. (2015).

Both Yang et al. (2011) and Sakon et al. (2010) reported the addition of inpatient pharmacological prophylaxis to reduce the risk of VTE compared to mechanical only prophylaxis (0.72% compared to 0.91% (Yang et al. 2001) and 1.2% compared to 19.4% (Sakon et al. 2010)). The large difference in VTE rate reported in Sakon et al. (2010) could be attributed to its broad inclusion criteria for operative type—including any laparotomy with curative intent for a malignancy, whereas Yang et al. included only colorectal cancer patients.

One study—Beyer et al. (2009)—included subgroups of asymptomatic and symptomatic DVT, reporting extremely high rates of asymptomatic VTE at both day 8 (96.5%) and day 21 (88.7%) postoperatively.

Regarding safety, only Sakon et al. (2010), Jeong et al. (2010), and Schmitges et al. (2012) reported safety outcomes associated with postoperative heparin VTE prophylaxis. Sakon et al. (2010) and Jeong et al. (2010) compared inpatient heparin with mechanical only or no prophylaxis, while Schmitges et al. (2012) compared 4 weeks of therapeutic dose heparin (60+ mg enoxaparin) with 4 weeks of prophylactic dose heparin (40 mg enoxaparin subcutaneously daily).

Unsurprisingly, Sakon et al. (2010) and Jeong et al. (2010) both report total bleeding events to be greater in patients receiving pharmacological prophylaxis (total bleeding complication rates of 9.17% and 13.4% in patients receiving heparin compared with 7.89% and 5.5% in those receiving mechanical and no prophylaxis). Sakon et al. (2010) also reported major bleeding events (defined as death, transfusion of more than two units of red cells, hemoglobin drop of more than 2 g/dL, or retroperitoneal, intracranial, or intraocular bleeding resulting in serious or life-threatening events) occur at a rate of 4.6% compared to 2.6% in those patients receiving mechanical prophylaxis with inpatient pneumatic compression alone.

Likewise, reported transfusion rates were lower when mechanical prophylaxis alone was used compared to those patients receiving additional pharmacological prophylaxis (Jeong et al. 2010; Schmitges et al. 2012).

In summary, analysis of the included studies did not identify any statistically significant reduction in postoperative VTE risk associated with extended duration use of heparin VTE prophylaxis compared to inpatient only duration prophylaxis. Nor was any statistically significant difference in rates of bleeding complications identified between the two groups. However, multiple smaller cohort studies not suitable for inclusion in statistical analysis report lower rates of postoperative VTE in patients receiving extended duration pharmacological prophylaxis, albeit with an increased risk of bleeding complications and transfusion requirement.

## Discussion

Overall, literature review of recently published papers (< 10 years old) exposes a surprisingly low level of poor-quality evidence for extended pharmacological VTE prophylaxis.

Two pre-existing systematic reviews were identified; however, only one study (Bergqvist et al. 2002) is included in both.

These are interesting to compare to the outcome of our study in that their outcomes are almost directly contradictory—while Fagaranasu et al. (2016) conclude that “extended thromboembolism prophylaxis significantly reduces the overall incidence of VTE and proximal DVT without increasing the risk of major bleeding,” Akl et al. (2008) is more consistent with our own results reporting “limited and low-quality evidence that extended duration LMWH for perioperative thromboprophylaxis reduces DVT in patients with cancer undergoing major abdominal or pelvic surgery” concluding that “more and better quality evidence is needed to justify extended regimes.” Criticisms of the Fagaranasu et al. review which may explain its conflicting outcome include the comparison of laparoscopic and open studies (i.e., dissimilar populations) and invitation of bias by including observational studies as well as randomized controlled trials.

It is also interesting to note the number of narrative reviews that we excluded on full text review. It is possible this volume of narrative reviews reflects other prior attempts to perform systematic review and meta-analysis in this area, where the poor level of evidence available precluded systematic review from being conducted.

A limited number of papers were appropriate for direct comparison, and these results must be interpreted with caution due to the small number of studies included, small sample sizes in some studies, and significant degree of heterogeneity observed between included studies. In addition, the population of the Samama et al. (2014) study was much larger than that of the other included studies and so the combined results need to be interpreted with an awareness of the impact of this on the overall outcome.

Allowing for this, our analysis found no significant difference in postoperative VTE rates or postoperative bleeding complication in patients receiving extended duration prophylaxis compared to those receiving inpatient duration prophylaxis.

However, on narrative review of the remaining literature unsuitable for direct comparison, many studies did observe cohorts receiving extended duration pharmacological prophylaxis to have lower rates of postoperative VTE and an increased rate of bleeding complications and number of blood transfusions.

Across all included studies, total rates of VTE events that were sufficiently symptomatic as to trigger investigation and objective confirmation of diagnosis by imaging were generally low. This may imply that clinically significant postoperative VTE is a rarer complication than many clinicians believe.

Assuming this were true, extrapolation would suggest that while pharmacological prophylaxis with heparin may convey a benefit, this benefit is likely small, and thus, the number needed to treat (NNT) would be high, therefore leading to a high cost of prophylaxis at a population level despite the individual cost being low*.* Thus, Kakkar et al. had only 2 patients in the extended group and 3 in the inpatient group that developed a PE (Kakkar et al. 2010). Samama et al. had 5 in the extended group and 10 in the inpatient group (Samama et al. 2014). Combining the 2 extended populations, one could conclude that extending VTE prophylaxis results in 6 fewer PEs for a total of 2620 patients who received extended prophylaxis. Given the small number of studies and small sample sizes of the articles included in our direct comparison, it is possible that a benefit does exist, and the comparison was simply underpowered to confirm this. If this is true, it is possible that the benefits of pharmacological VTE prophylaxis in reducing postoperative VTE may in fact be outweighed by the financial cost of treatment and/or potential complications associated with its use. A cost benefit analysis was beyond the scope of the present study.

In contrast, when asymptomatic VTE was considered, this appears to have an extremely high prevalence postoperatively, with some studies reporting up to 96.5% on day 8 and 88.7% on day 21 postoperatively (Beyer et al. 2009). If this is assumed to be true, with a prevalence this high, it would appear unlikely it carries any truly significant implication on overall patient outcome, and raises the question if it is possible asymptomatic DVT falls within the spectrum of normal physiology following a traumatic insult to the body such as major surgical intervention in the abdomen or pelvis.

This outcome, while unexpected, does raise important questions. Firstly, the use of pharmacological VTE prophylaxis is an established practice worldwide, and while there is a lack of consistency between type and duration of prophylaxis used by individual providers and centers, extended duration is becoming extremely common. In failing to identify any significant reduction in VTE risk associated with extended duration prophylaxis, we must wonder how this became an established and widely accepted part of clinical practice without a truly strong evidence base.

While there is certainly evidence for its use in other specialties [23], it is uncertain to what extent it is appropriate to apply evidence from other specialties such as orthopedics to patients undergoing pelvic surgery. While patient characteristics are likely to have commonalities across both specialties, vastly different operative characteristics and the presence or absence of malignancy in the patient result in a significant difference between the populations.

Limitations of our study included the small number of identified studies suitable for analysis and the small sample sizes of included articles. This could be improved by extending the time period used in the study’s inclusion criteria to include articles older than 10 years. In addition, due to the limited number of suitable articles identified, some included papers do include some data not exclusively from patients undergoing open surgery.

## Conclusion

In conclusion, our study found no significant difference in postoperative VTE rates or bleeding complication in patients receiving extended duration heparin VTE prophylaxis compared to those receiving inpatient prophylaxis after open abdominopelvic surgery for malignancy. However, the available evidence was limited and of poor quality, so this finding must be interpreted with caution.

This result raises the important question of how the use of extended duration prophylaxis in this population has become widespread, common practice without a truly strong evidence base proving a benefit. If this is truly the case, we question whether current recommendations regarding its use should be re-evaluated.

## Data Availability

Data used and analyzed in this review are available from the corresponding author on reasonable request.

## Abbreviations

AUA: American Urological Association
BJUI: *British Journal of Urology (International)*
CTP: Conventional thromboprophylaxis
CTPA: Computed tomography pulmonary angiogram
DVT: Deep vein thrombosis
ETP: Extended thromboprophylaxis
ICU: Intensive care unit
LMWH: Low molecular weight heparin
NHMRC: National Health and Medical Research Council
NNT: Number needed to treat
PE: Pulmonary embolism
RCT: Randomized controlled trial
US: Ultrasound
V/Q: Ventilation/perfusion
VTE: Venous thromboembolism
